# Self-reflection as the metacognitive “rubber band” for psychologists in practice: a qualitative exploration

**DOI:** 10.1080/00049530.2025.2568762

**Published:** 2025-10-13

**Authors:** Stephanie E. Banner, Adam J. Rock, Suzanne M. Cosh, Nicola Schutte, Kylie Rice

**Affiliations:** School of Psychology, University of New England, Armidale, Australia

**Keywords:** Self-reflection, metacognition, self-awareness, introspection, reflective practice, reflection

## Abstract

**Objective:** Self-reflection is widely recognised as an essential skill for health professionals across the world. Models and accreditation standards highlighting psychology competencies have shared the view that self-reflection sits alongside other competencies such as assessment and intervention. However, there is limited research to date exploring the experience of self-reflection for psychologists, particularly research through a cognitive and metacognitive lens. This study aimed to explore the cognitive and metacognitive processes, and barriers, for psychologists when engaging in self-reflection.**Method:** Reflexive thematic analysis with a hybrid inductive/deductive approach was used to analyse 12 interviews with psychologists who shared their experience of self-reflection.**Results:** The analysis identified an overarching theme of interaction between the personal and professional self, and five subthemes of 1) attentive and responsive to cues, 2) personal and contextual barriers to self-reflection, 3) intentionally overcoming barriers, 4) meeting immediate motivations and 5) reflection for learning.**Conclusion:** This research adds to the current literature on self-reflection, with added depth of understanding through a cognitive and metacognitive lens, and provides evidence for the applicability of elements of existing models of self-reflection to psychologists.

## KEY POINTS

**What is already known about this topic:**

1. Self-reflection has been recognised as crucial for effective clinical practice across health disciplines, including applied psychology (e.g. Cooper & Weickowski, 2017; Knapp et al. 2017; Prasko et al. 2012).
2. Self-reflection is thought to play a role in the broader professional development of psychologists and interacts with other psychological competencies for continued skill development (Rodolfa et al. 2005).
3. Self-reflection is also believed to facilitate therapy through the development and maintenance of a strong therapeutic relationship (Prasko et al. 2012) and to assist practitioners with navigating clinical decision-making (e.g. Lilienfeld & Basterfield, 2020).

**What this topic adds:**

1. Qualitative interviews and reflexive thematic analysis with a hybrid inductive/deductive approach allowed for rich exploration of self-reflection with psychologists.
2. Psychologists shared that self-reflection is characterised by an intersection of the personal and professional self, and that self-reflection is a useful tool both in immediate practice and for overall personal and professional development.
3. Psychologists described a process of engaging in self-reflection which had considerable overlap withe xisting models of self-reflection, however also discussed personal and contextual barriers and how they overcame these which is not otherwis eencapsulated in the existing models of self-reflection.

Reflection is widely discussed and frequently defined (e.g., Bennett-Levy et al., 2009; Nguyen et al., 2014) and is a mandated competency of psychology in several countries (i.e., Australian Psychology Accreditation Council, 2019; British Psychological Society, 2024). Just over 20 years ago, Bennett-Levy (2003) noted that “reflection hasn’t just had poor press in psychology. Over the past 60 or so years, it has had almost no press at all” (p. 16). Twenty years later, the value of reflection has been widely reported in the literature related to psychologists and the psychology profession, with the suggestion that it can positively impact other psychologist competencies such as initial skill acquisition (Bennett-Levy, 2006), cultural responsiveness (Smith et al., 2021) and the continued development of other competencies (e.g., assessment) beyond registration and across the career trajectory (Rodolfa et al., 2005).

Early links between reflection and learning were made by Dewey (1933), who proposed that it is not merely experience that leads to learning, but reflection on these experiences. Schön (1983) extended Dewey’s work to psychotherapists and described the way in which it is possible to simultaneously engage in reflection and the task at hand (reflection-in-action). Killion and Todnem (1991) elaborated on Schön’s (1983) work further to describe reflection-for-action, whereby reflection can be used for planning and change in the future. This is a commonality in a number of other models of reflection, such as Kolb’s (1984) experiential learning theory, Gibbs’s (1988) reflective cycle, Lawrence-Wilkes and Ashmore’s (2014) mnemonic REFLECT model, Grimmer’s (2022) cloverleaf model and Zimmerman and Campillo (2003) model of self-regulated learning. Rolfe et al. (2001) summarised and simplified commonalities in the literature in their model of reflective practice, wherein one considers the event and its meaning and then steps through the prompts of “what, so what, and now what” (p. 35). These models all share an emphasis on reflection as a mechanism for learning from experience and then applying changes.

Definitions of reflection continue to vary across the literature (Nguyen et al., 2014) and are often conflated with similar yet distinct concepts such as reflective practice, self-reflection and reflexivity. Reflective practice definitions typically situate reflection and self-reflection within professional practice (e.g., Fouad et al., 2009), with some authors describing reflective practice as the formalisation of self-reflection for practitioners (Lilienfeld & Basterfield, 2020). Self-reflection has been identified as a “deliberate metacognitive process” (Knapp et al., 2017, p. 167), which “leads to insight and change in one’s behaviour or attitudes” (Knapp et al., 2017, p. 167). Bennett-Levy et al. (2009) summarise the distinction between reflection and self-reflection:

Reflection is the process of intentionally focusing one’s attention on a particular content; observing and clarifying this focus; and using other knowledge and cognitive processes (such as self-questioning, logical analysis and problem solving) to make meaningful links. Self-reflection is a specific form of reflection in which the content for reflection is self-referenced to one’s thoughts, feelings, behaviours or personal history.

(p. 121)

Self-reflection shares commonalities with reflexivity, which highlights an awareness of social and cultural factors as part of self-reflection (Coulson & Homewood, 2016) and how these shape practice (Psychology Board of Australia, 2024). Reflexivity is also understanding how behaviours impact sociocultural norms and structures (Bolton, 2010) and is applied to research contexts to understand the positionality of the researcher and how this impacts the research (Braun & Clarke, 2022; Finlay & Gough, 2003). There is also emerging conceptualisation of terms such as metacognitive reflection, which describes the way in which metacognition is applied to the process of reflection (Merkebu et al., 2024), answering calls for the integration of metacognition into the reflective practice literature (e.g., Eva & Regehr, 2005; Lilienfeld & Basterfield, 2020).

The link between self-reflection and learning has been formalised in the declarative-procedural-reflective [DPR] model (Bennett-Levy, 2006; Bennett-Levy et al., 2009). The DPR model posits that practitioner growth is the result of the development and interaction of three systems: declarative, procedural and reflective systems. This distinction is informed by earlier work by Binder (1993, 1999), who stipulated the difference between declarative knowledge – or factual information – and procedural knowledge – or the “how to” and “when to” (p. 59) of the declarative information. For example, declarative knowledge may detail the steps involved in a graded exposure intervention, whilst the procedural knowledge indicates how and when to complete the specific steps. The reflective system is metaphorised as “the ‘engine’ which drives lifelong learning as a therapist” (Bennett-Levy et al., 2009, p. 119) and is central to the DPR model, impacting all other elements. Bennett-Levy et al. (2009) describe the reflective system as having three steps: focused attention on a problem (which may be in relation to practice, the practitioner, or both), reconstruct and observe, and then conceptualise and synthesise. In this way, the reflective system acts as a tool for therapists’ ongoing learning.

The value of self-reflection in procedural and declarative learning has been formalised in self-practice/self-reflection (SP/SR) models of teaching and learning (Bennett-Levy et al., 2001). SP/SR has been found to increase technical and interpersonal skills (Davis et al., 2015) and wellbeing, resilience and reflective skill (Mösler et al., 2023). The Personal Practice [PP] model (Bennett-Levy & Finlay-Jones, 2018) extends the DPR model to provide a theoretical framework to account for the way in which SP/SR impacts professional development. A core component of the PP model is the “reflective bridge” (Bennett-Levy & Finlay-Jones, 2018, p. 191), whereby practitioners engage in three processes of self-reflection: personal self-reflection, therapist self-reflection and the reflective bridge which serves to synthesise personal and therapist self-reflection, and facilitates skill development in SP/SR training modalities. The PP model has been supported through qualitative analysis of reflective statements from trainee therapists which identified that engaging in SP/SR supported personal development, which ultimately fostered a greater understanding of the self and the way in which this shapes therapy, experience of therapeutic techniques and subsequent belief that these insights and experiences would positively impact their professional practice (Presley & Jones, 2024). This is further evidence of the utility of self-reflection in training and development.

An assumption of existing definitions and models of self-reflection is that the introspection of thoughts, feelings and actions can be a helpful process for psychologists. However, as the broader cognitive behavioural literature shows (e.g., Beck, 2020), thoughts, feelings and actions can occur in maladaptive ways that are at odds with overall goals and motivations. Such barriers to self-reflection have received little attention in the literature, perhaps partially attributable to the difficulty in operationalising self-reflection. Further, as self-reflection can have a metacognitive component (Bennett-Levy, 2006) – in that the process of self-reflection (rather than the triggering experience that was the initial stimulus for self-reflection) may also be evaluated as it occurs – there are questions as to how psychologists perceive self-reflection and what the process involves for them.

Further, the literature on self-reflection continues to be afflicted by the unique difficulties that such a directly unobservable phenomenon raises. As Bennett-Levy (2003) noted, “reflection is a behavioural scientist’s nightmare – almost impossible to define tightly, and well nigh uncontrollable” (p. 16). There has been a dearth of validated, structured measures of self-reflection for health practitioners more broadly (Ooi et al., 2020) and even less so for psychologists specifically (Banner et al., 2023). This results in difficulties in testing and validating models of self-reflection. Qualitative research has provided some insight, with Fisher et al. (2015) finding that Singaporean psychologists assigned considerable value to reflection, though the researchers also noted the difficulty psychologists found in describing reflective practice and specifically how they engaged in this process.

## Objective

The present study aimed to further understand the nature of self-reflection for psychologists from a cognitive and metacognitive perspective. This examination is a expansion of the current literature, as the applicability of existing models of self-reflection have not been explored with psychologists.

The present study used a qualitative design to explore the following research questions:

1. What are psychologists’ experience of cognitive and metacognitive processes to engage in self-reflection?
2. What are the perceived cognitive and metacognitive barriers to engaging in self-reflection?

## Methodology

### Research paradigm

The research was conducted within a critical realist paradigm, acknowledging that participants are sharing their version of reality which is then interpreted through the lens of the researchers (Braun & Clarke, 2022). The researchers all possess a background in psychology academia and/or practice and consequently were familiar with the concept of self-reflection and had experience in personally engaging in self-reflection as part of reflective practice. Reflexivity allowed the researchers to apply this knowledge and experience in a meaningful way throughout the study. Reflexive thematic analysis (Braun & Clarke, 2022) guided the research design, including methodology and data analysis, as it allowed for the use of researcher self-reflection alongside that of participants to generate meaningful data and analysis.

Critical realism emphasises the value of theory (Willis, 2022). This also aligned with the research objectives to understand the experience of self-reflection for psychologists and how this aligns with existing models of self-reflection. A hybrid inductive/deductive approach to coding and thematic development was used, capitalising on both the researchers’ empirical knowledge of self-reflection and participants’ lived experience of self-reflection. A hybrid deductive/inductive analysis has the benefit of being data-informed, allowing for rich analysis whilst also drawing on relevant theories to inform analysis (Proudfoot, 2023). Hybrid deductive/inductive analysis can be suitable where theories and models are of relevance but do not fully capture the data (Proudfoot, 2023). This approach was chosen because it was expected that there may be some overlaps with existing models of self-reflection though also that participants would offer a unique perspective beyond the current models. It is also thought that this approach may increase the rigour of the analysis (Fereday & Muir-Cochrane, 2006).

### Design

A qualitative research design using interviews was selected to allow for the rich data collection and analysis required in the context of the current research questions. Rather than adopting a rule-of-thumb approach to sample size, information power was used as the metric for sample size. Information power refers to the depth of information, rather than its volume (Malterud et al., 2016, 2021), for facilitating rich and meaningful analysis. Information power (Malterud et al., 2016, 2021) required participants to be comfortable and willing to share their understanding and experience of self-reflection, and so interviews were chosen over other qualitative methods in order to increase information power through maximising rapport and confidentiality to facilitate participants feeling comfortable to share experiences. Further, the lead author conducted all interviews and advised participants of their background in psychology research and practice. This aided the depth of information in the interviews in various ways, including rapport building and assisting participants to use jargon without needing to provide unnecessary consideration to explaining the meaning to the interviewer. This depth of information combined with the specific research aims and relatively homogenous sample (as practicing psychologists) led the authors to conclude that the current sample would provide sufficient information power for meaningful analysis.

#### Participants

The study was announced in an online advertisement and was promoted alongside similar research on the University website and across various professional platforms such as LinkedIn and psychology professional events. Participants were included in the study on the basis of holding current Australian registration as a psychologist. Inclusion criteria were intentionally broad to maximise recruitment and capture psychologists working across various practice settings and geographical areas. Psychologists were offered a notional gift card for partaking in the research. Twelve psychologists expressed interest in participating in the research and all completed the interview (See Table 1). Nine participants were female, and three participants were male. Participants had a range of experience, spanning 0–5 years of registration up to 30–40 years of registration, with half of the participants having held experience for 0–10 years. Participants also had experience across a range of practice settings and clinical populations.

**Table 1.** Interviewee characteristics.

Participant	Sex	Years of registration	Practice Context
1	Female	20–30	School Psychology
2	Male	20–30	Aboriginal Medical Service
3	Female	0–5	Public Health (Child and Adolescent) and Private Practice
4	Male	6–10	Government (Child Protection)
5	Female	10–20	Public Health (Child and Adolescent) and Private Practice
6	Female	6–10	Private Practice
7	Female	6–10	Government (Justice) and Private Practice (across the lifespan)
8	Male	0–5	Private Practice (Individual & Groups)
9	Female	6–10	Public Health (Child and Adolescent)
10	Female	30–40	Private Practice (across the lifespan)
11	Female	10–20	Academia with recent experience with Children and Adolescents
12	Female	6–10	Private Practice (across the lifespan)

#### Procedure

All participants first provided informed consent through the Qualtrics^TM^ (https://www.qualtrics.com/en-au) online survey service. Interviews were conducted either in person or via Zoom teleconferencing software based on the participants’ geographical location and preference and recorded for later transcription and analysis. Participants were asked a number of questions about self-reflection, both generally (e.g., “*Tell me about your role as a psychologist and how you see self-reflection fitting with this*”) and more specifically (e.g., “*How often do you participate in self-reflection activities?*”, “*What do you use self-reflection for?*” and “*How does engaging in self-reflective activities help you as a practitioner?*”). Whilst reflexivity fostered researcher awareness of their own definition and experience of self-reflection, the interview was structured with the initial questions being quite broad so as to facilitate participants sharing their own meaning and process of self-reflection. This approach also increased information power. Probes and summaries were used to further prompt sharing from the participants. Interviews spanned 45 to 75 minutes.

### Data analysis and reflexivity

Analysis was conducted in accordance with the six phases of reflexive thematic analysis proposed by Braun and Clarke (2022). This process was iterative, with multiple theme and coding revisions throughout the process. The first author transcribed all recordings verbatim before commencing coding in NVivo (version 12; Lumivero, 2017). NVivo was used as a database, allowing the first author to review and refine the codes across the dataset throughout the coding process. Specifically, coding by the first author occurred in multiple stages, with transcripts being initially coded in random order once all interviews had been completed. Codes were then reviewed both through subsequent review of the transcripts and through examining the codes as an entire list and identifying where codes may be redundant due to overlap or insufficient meaning. This process of reviewing codes included itemising them according to frequency; however, whilst codes were considered for removal if they were not identified many times across the transcripts as they may have been too specific (Braun & Clarke, 2022), if such a code was considered meaningful and not better captured by more prominent codes, then it was retained. Whilst coding started with an inductive approach, codes were then reviewed to ascertain overlap with existing theories. Models that particularly informed coding included Schön’s (1983) conceptualisation of reflection-in-action, reflection-for-action (Killion & Todnem, 1991), and more cyclical models of self-reflection (e.g., Kolb’s, 1984 experiential learning theory; Gibbs’s, 1988 reflective cycle; Lawrence-Wilkes & Ashmore’s, 2014 REFLECT model), which share an emphasis on reflecting on past practice to elucidate new insights and consequently improve future practice (Lilienfeld & Basterfield, 2020). This was an iterative process with back-and-forth between inductive and more deductive, theory-led coding. Coding also became more latent and less semantic as researcher reflection continued throughout the process of refining codes; however, codes were also then reviewed in the context of the broader transcript to ensure that the latent interpretation of the text matched the context, in an effort to ensure a balance between participant contribution and researcher reflexivity that maintained the integrity of the data. Codes were then clustered into groups based on shared meaning, whereby some codes were removed in favour of other codes that better captured the construct. Relationships between the codes and themes were then diagrammatically presented by hand across numerous iterations. These diagrams were refined into thematic maps over time in consultation with the other authors over numerous instances of peer debriefing. Researcher reflexivity was crucial in the process of coding and theme refinement, in terms of balancing unique participant contributions and recognising where participant contributions matched existing models. Themes were revised multiple times throughout the construction of the manuscript.

Trustworthiness was considered throughout the research design, execution, analysis and reporting (see Nowell et al., 2017, for review). Particularly pertinent to the design, execution and analysis was engagement in reflexivity, including through the use of a reflexive journal by the first author. Key elements of the reflexive journal were shared with the research team through peer debriefing (see Janesick, 2015). As an insider researcher, the reflective journal assisted with considerations such as reflecting after interviews about how the interviewer’s knowledge and experiences of self-reflection may have shaped what participants shared (e.g., areas where the first author may have made assumptions, or may have prompted more or less for further information). Further, the iterative process of code development and refinement, as well as theme development and refinement, was reviewed with the research team at various stages. In the context of the hybrid inductive/deductive approach used for analysis, this also included discussions around balancing the unique contribution of participants alongside the value of existing theories in developing meaning from the data. Again, as an insider researcher, reflexivity was crucial for the first author in questioning the extent to which code and theme development was data-driven, rather than researchers transposing their own experience of self-reflection onto the data. In reporting the research, the research team reviewed the manuscript to ensure sufficient detail was included to assist readers with determining transferability.

## Analysis

**Figure 1.**
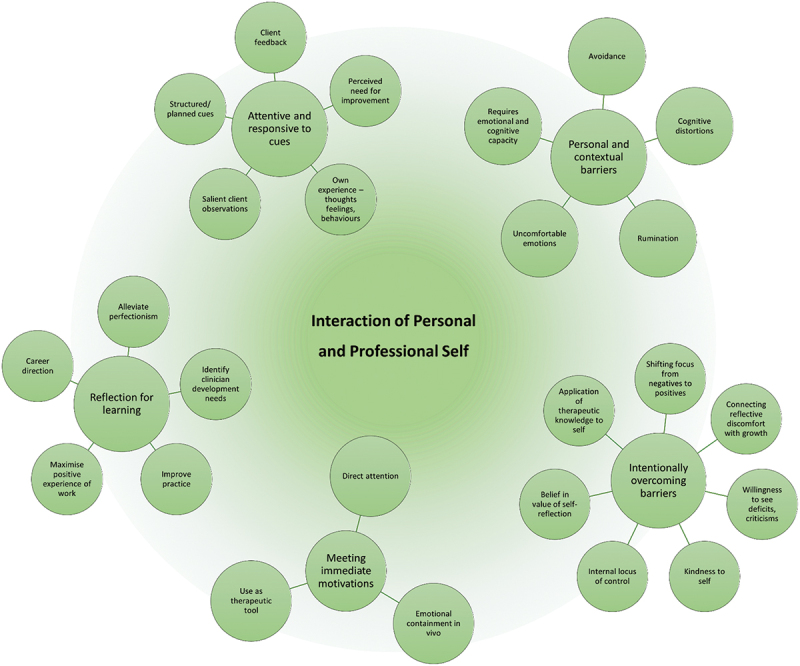
Thematic map: interaction of the personal and professional self.

Through analysis, codes were clustered into a thematic map that captured one overarching theme with five subthemes and their related codes (Figure 1). The overarching theme is interaction of personal and professional self with subthemes of 1) attentive and responsive to cues, 2) personal and contextual barriers to self-reflection, 3) intentionally overcoming barriers, 4) meeting immediate motivations and 5) reflection for learning. The overarching theme of “interaction of personal and professional self” is the common thread across the subthemes, with personal and professional motivations, barriers, strategies and learning all being a facet of self-reflection. Together, the subthemes highlight the way that psychologists use self-reflection as a tool to identify and attend to salient cues, identify barriers as they arise and then intentionally choose strategies to overcome them effectively, which facilitates both meeting immediate motivations (e.g., in session goals) and more broadly facilitating learning (e.g., personal and professional development).

### Interaction of personal and professional self

The overarching theme of interaction of personal and professional self identifies the way in which self-reflection is impacted by both the personal and professional selves across both earlier components of self-reflection (i.e., with motivation informing what cues are perceived as salient and require action), introspection (i.e., with personal barriers arising throughout the process and personal strategies are utilised to overcome these barriers) and then in the resulting behavioural change (i.e., as a means of meeting immediate goals in session, or as part of broader learning and development as a person and clinician).

#### Attentive and responsive to cues

Participants shared a sense of self-reflection as often being prompted by a cue of some form, whether this was an observable external event (e.g., a team meeting or peer supervision) or an internal event (e.g., noticing one’s own thoughts, physiological experiences and emotions). This was captured as a subtheme of being attentive and responsive to cues, as participants described not only noticing a cue in themselves or others but also responding by diverting their attention to the cue.

Participants described self-reflection as occurring in response to a self-based cue, such as noticing their own thoughts, physiological feelings or emotions. Although psychologists are often working with others, in terms of being in both the presence and service of others, psychologists use their own internal experience as a stimulus for self-reflection. For example, P5 described the role of their own feelings and the appraisal of those feelings in prompting self-reflection: “*immediate feeling of, like, oh okay, that was an uncomfortable conversation, and I really didn’t enjoy seeing that person. And then there’s usually that moment afterwards to just shake that off and notice that. And let it go*.” This indicated a sense of not only noticing internal cues but also how this can allow the practitioner to move on with their day. This sense of observing the self was further described by P10, who also described integrating this information with observations of the client, and the way in which this may also shape the practitioner’s behaviour in response to, first, the observation and, second, the resulting reflections:

Because if there was some kind of loss of equanimity or loss of equilibrium … that will show up in the body, your own body and the body of the other person, and then kind of use that sensory input to kind of look at what you’re thinking, what you’re feeling, what you just did, like the behaviours and … also the other person and then making kind of making adjustments.

These participants all shared a sense of noticing what may not be going well as a prompt for self-reflection rather than noticing positive responses in themselves or the client. Relatedly, participants also described that self-reflection may arise in response to a perceived need for improvement. P12 described a process of identifying a challenge and considering the next steps “ … of doing a bit of self-reflection of where do my competencies sit in this case, and, how can I work within my competencies, but also what skills do I need to improve?”

P12 further described a wider lens for self-reflection in terms of responding to the needs of their general client load and then using this to shape their professional development: “*self-reflection of what areas have I had a bit of a challenge with or … what … am I seeing a lot of at the moment that I’m feeling a little bit out of depth with and using … that to pick PD [professional development]*.” There was also a sense that self-reflection may be in response to client feedback. For example, P8 described questions that they ask themselves in response to experiencing some uncomfortable feedback: “*I think a sign of a competent psych is to step back from that and realise that what’s the grain of truth in that comment … ? What is the part of that I can actually grow from?*” Again, these comments indicate a sense that self-reflection may often be prompted by a perceived difficulty rather than in response to a positive event.

However, participants also at times described a more structured approach to self-reflection, with cues such as supervision, case note writing or scheduled time as part of a weekly routine. For example, P5 noted:

structured reflection relies on the meetings as a way to kind of prompt them. Or even sometimes I would possibly take a moment or take a walk before a meeting or prepare a written formulation or review, and that might be my way of just gathering my thoughts about something.

Case notes were also commonly described by participants as a cue for self-reflection; however, the nature of the self-reflection varied across participants. For example, P4 described engaging in self-reflection following note writing (“*I mean, you’ll write the consult note, then you’ll self-reflect, that being a more internal process rather than written*”), whilst P5 described this occurring during writing case notes (“*there’s my own personal … reflective practice which I think often tends to happen in note writing afterwards*”). P6 described the way in which case note writing can be a prompt for planning:

as I’m typing up the notes, I’m reflecting within that process as well, planning and thinking about questions as well, or maybe I need to do this but … I’m not taking notes specifically for reflection. I leave that for supervision.

This also indicates that participants are responding to cues in specific ways, in terms of choosing how or when they might engage in self-reflection according to the context. This suggests that, whilst self-reflection is responsive to cues, psychologists may also be making choices about the way they self-reflect based on the context.

#### Personal and contextual barriers to self-reflection

Within self-reflection, participants described a number of barriers related to the internal experience of self-reflection, with considerable overlap between cognitive and behavioural processes described by psychologists when engaging in self-reflection. Participants described barriers related to cognitions, metacognitions, emotions and behaviours.

For example, the experience of rumination and the discomfort that it accompanies was noted by a number of participants and commonly identified as unhelpful or “*problematic*” (P7). P9 linked this: “*anxious self-reflection: it definitely becomes ruminative*.” P8 further described this: “*maybe shame, sometimes maybe even guilt. Not for all but definitely for me. So I think the function of self-refection needs to be kept as that and being able to define self-reflection as separate from unhelpful ruminative self-punishing*.” This conveys a sense of the relationship with rumination and the way in which psychologists may be thinking of self-reflection as a cognitive process in and of itself and seeking to differentiate it from other cognitive processes, such as rumination or other unhelpful cognitive patterns. In a similar vein, cognitive distortions were also identified as a barrier by participants. For example, P8 described that “*self-reflection can be a door to self-punish*” and that it is therefore necessary to “*be mindful of black-and-white ‘oh I’m bad now’; any of the personal stuff coming up*”. There was a sense that these cognitive distortions were an interaction of the “professional” and “personal” selves, with distortions around the self impacting self-reflection in a professional context. P7 also noted the way that their mood can influence their self-reflection, specifically through a bias for noticing the negatives: “*if you’ve had a big day, and it’s been heavy, it’s easy to focus on the things you’ve done wrong*.” This also indicated a sense that, at times, one may not have the cognitive capacity to effectively engage in self-reflection. P12 identified the way in which this may manifest as imposter syndrome: “*[self-reflection] fuels a bit of imposter syndrome in terms of ‘oh I … should have done that better’ and all those sorts of things*.” It was also noted that, due to the demanding nature of self-reflection, one must have the “*bandwidth*” (P8) to be able to engage with it, as “*if you’re already a bit beaten down and worn down and burnt out then I don’t think that it’s always effective to self-reflect*” (P8). P8 extrapolated that it can be difficult to accept the need for change that self-reflection can sometimes bring: *“It’s like I can’t deal with the possibility that I’ve got to work a bit harder … I’m just too down on myself right, like, I can’t do this … right now*.”

In the context of these uncomfortable thoughts and feelings, participants noted that avoidance can be a barrier to self-reflection. Avoidance is recognised across cognitive behavioural therapies as a response to uncomfortable thoughts and feelings (e.g., Harris, 2019; Hayes et al., 2012). For example, P8 articulated, “*So, what I’ll end up doing is, I’ll do some avoidance or gentle avoidance in supervision, I’ll start talking about process, about my own self-reflection*.” This participant noted that talking about the way in which they self-reflect can, in fact, be avoidance of self-reflection itself, indicating metacognitive awareness of the process of self-reflection

Time was also noted as a barrier to engaging in self-reflection and how this may be shaped by context, such as roles and workplaces. Consideration was given by the researchers as to whether this reflected a more latent meaning of avoidance in the face of uncomfortable thoughts and feelings as described above; however, it was unclear from the transcripts as to whether time was cited as a barrier more in the context of uncomfortable thoughts and feelings. P3 noted that it was easier to engage in self-reflection in the public sector rather than in private practice: “*I tend to have more of an opportunity to do self-reflection in my public health work just because of having time in the day where there aren’t clients every hour*”. P5 reported that other tasks, “*tend to take priority*.” Despite the fact that self-reflection is a competency sitting equally alongside other psychological competencies, such as assessment and intervention (e.g., Rodolfa et al., 2005), self-reflection appears to be a lower priority for some clinicians on occasions where time is limited.

#### Intentionally overcoming barriers

Whilst self-reflection may be uncomfortable at times, participants also described a number of strategies to overcome these barriers. Participants described a number of cognitive strategies, such as shifting focus to what may be going well rather than deficits. Whilst participants frequently reported asking themselves questions about what they could improve (as discussed above regarding attending and responding to cues), which aligns with a deficit-based approach to improving practice, some participants articulated the value of also noticing what went well. Participants shared barriers relating to focusing on the negatives of their practice, with P8 describing how they must consciously shift their attention to identify strengths and positives as well:

My schemas lend themselves to “what can I do better, what have I been doing wrong?” And you’re right, it’s an extra layer: well, hang on, what am I doing well? And that’s probably a bit more uncomfortable for me to do.

This also touches on the intersection of the personal and professional self in self-reflection, with this participant drawing on their own knowledge of therapies to identify their cognitive patterns and then adjust these to better facilitate self-reflection. Participants spoke using therapy language (e.g., P10 also spoke of mindfulness); however, these differed across participants, suggesting that some psychologists engaged in strategies that held particular value to them.

Participants also described helpful metacognitive processes, in terms of how they first monitor and notice and then use the cognitive strategy of reframing, through a more positive, constructive lens, the discomfort and unhelpful cognitions arising through self-reflection. P8 used the metaphor of a “*rubber band*” to describe the way in which they were “*stretched*” by self-reflection, which can be:

pretty painful … it feels like you’re stepping back, but the only difference is that you’re aware of your areas to grow, and that’s the difference, so sort of wool over your eyes before then, and then you feel like you’ve stepped back, but the only difference is you’re aware of it. And then sort of balancing it with I know stuff, and there’s more stuff to know.

This indicates a sense of not just noticing one’s thoughts and feelings in self-reflection but also then challenging the accuracy of any negative cognitions that may be unhelpful, and how this ultimately fosters a sense of acceptance of the dialectic of having knowledge and needing to acquire further knowledge.

Whilst participants shared their sense of challenging unhelpful cognitions, there was also a sense of needing to engage in criticisms without deflection. This was captured as a willingness for criticism, including, according to P9, “*being able to be critical in that process in a helpful way*” and, according to P2:

there are some things I can do better, some things I might reflect back on and think I didn’t do that as well as I would like to have done. And I think that that’s important for me to be genuinely but constructively critical of myself. And I think reflexivity helps me to do that.

However, there was a need to balance criticism with kindness, as P12 described: “*I need to get better at being a bit more honest and objective with myself, but also kinder to myself as well*.” This echoed other comments regarding a metacognitive awareness of self-reflection, in terms of participants understanding how they could engage in self-reflection in a more effective way. This may also overlap with using one’s own therapeutic knowledge (e.g., in self-compassion) to overcome barriers to self-reflection.

Participants also described a number of strategies that may prevent rumination: for example, focusing on factors that could be controlled or changed. P3 described identifying their locus of control as an effective way of self-reflecting on a situation: “*it ended up being a situation where there’s a communication breakdown, and it wasn’t only my fault, however … it’s thinking, okay I had a part in this. What can I do, as I can only control how I respond*.” P7 also identified focusing on things that could be changed in the present:

Ruminating … I think where it can become problematic is where you aren’t seeing that client anymore, like they’ve dropped out of treatment, and you’ve heard it on the grapevine from another colleague or something, and you think, “oh, I should have done that differently”, and that’s where it can become problematic, like a bit of a loop: “Oh why did that client not come back?” And just, I think in order to stop that in its tracks, it’s giving it that space, but also what can I do differently with my clients I have now? What can I do in the future? Where can I build that into therapy?

P7 highlighted “*giving it that space*”, which also indicates the role of tolerating the distress rather than avoiding it, which becomes a barrier to self-reflection. Distress tolerance was also fostered by P8 through reminding themselves of the utility of self-reflection:

I remember my colleague that’s really experienced, and she said, if you ever get to a spot where you’re really comfortable, you’ve got to look within, and actually that’s making more sense now speaking about it … because you’ve settled, and you’re not growing anymore and maybe you’re not being as reflective.

Overall, there was a sense that psychologists were using metacognition to monitor self-reflection by engaging in introspection, and then engaging cognitive and emotional strategies to overcome barriers. Whilst this was common across participants, the specific strategies being used were sometimes unique to participants, indicating the role of self-awareness in identifying the most appropriate strategies to overcome barriers to effective self-reflection.

#### Meeting immediate motivations

Self-reflection was commonly discussed as a cognitive process which therapists were engaging in in the moment, akin to Schön’s concept of reflection-in-action. Self-reflection was discussed as a tool for meeting immediate motivations in the context of appointments with clients. P10 described the way in which reflection and attention direction is an iterative process which acts as a tool for the clinician in session:

I think, for the moment-to-moment kind of situation, reflection is really important because we need to reflect and then pay attention and then reflect on what you observed and then adjust accordingly. So that’s a moment-to-moment kind of thing, and a lot of that will be done – I don’t mean every single microsecond – but, you know, frequently. Especially when you get cues from the other person that, you know, looking at what’s happening there, observing what’s happening, and then reflecting on the situation.

This indicates a sense of monitoring in the context of the motivations for that session, such as monitoring clients’ reactions to ascertain the effectiveness of the session thus far. There was a sense of self-reflection aiding the containment of the emotional component of the work for psychologists, such as countertransference. Working as a psychologist may be an emotionally stimulating experience given the emotional content of the stimuli psychologists are engaged with throughout the course of the session and workday. Participants described how, if in an appointment with a client, the psychologist notices feeling emotional arousal, self-reflection can be a useful tool for re-establishing goals and directing behaviour accordingly. For example, P3 described an instance of countertransference they had experienced earlier in the day:

Another thing that came up today in my self-reflection was recognising that I was responding … to the dyad I was seeing between the young client and the parent, and I was able to reflect that … my own relationship with one of my kids was in my head as I was becoming frustrated with this client, and so that was actually a really, really useful use of self-reflection … being able to tweak that and then identify … so recognising that watching them is triggering me because of something that my own kid is up to … it’s then, okay, right, so what’s the action … what did I actually do in the moment, what else could I do, and is my emotional reaction getting in the way … ?

This suggests that participants used self-reflection, as a means of managing emotional responses so as to continue engaging in the session in a productive and safe way. Again, this shares the common theme of being driven by the psychologists’ personal and professional motivations in this moment: to continue psychological practice in the moment as effectively as possible. This was further touched on by other participants (P6 and P8) in the context of the value of personal therapy, with P6 noting the value of this in assisting them to “*not be reacting … based on my own issues*”.

There was a sense that the insights gained from self-reflection could be shared with clients as a therapeutic tool to add value to the session. For example, P5 described how they might use their own thoughts, feelings and decision-making with the client in response to what the client has shared with them:

I do use it as a tool for some clients, so some presentations, I find it helpful that there’s a lot more transparency in my process in the sessions. So I might share when something they share triggers a thought or triggers an experience for me as a way of, I guess, helping with that mentalising process for them … it’s just helpful for them having “okay, well these are the thoughts that I’m having about what you’re telling me and this might be what I can do with that, but you know happy to discuss”. So there’s definitely reflection that’s happening in the space and … sharing that as a tool.

Sharing select self-reflections in session is an overt use of self-reflection as a tool to assist psychologists in meeting their goals for the moment.

#### Reflection for learning

Participants described engaging in reflection after the fact and described a broad range of outcomes that motivated this engagement in self-reflection, beyond those described in relation to reflection in the session. For example, participants described that self-reflection could also facilitate the identification of development needs. This can be preceded by identifying a gap between current practice and desired practice. P5 described how this occurs for them:

[Self-reflection] helps me see where I need to maybe do something differently … it helps me reflect on what is working for the client and what probably isn’t working, what I did that maybe didn’t work so well and then think about doing it in a different way, the skills I need to learn that maybe I don’t have yet. So that’s … that prompting for more professional development.

This was closely linked to the value of self-reflection in developing their skills as a practitioner. For example, P1 described the relationship between self-reflection and their “*currency*”: “*It just means that I’m just over so many more topics and that reflection process on my skills is so enmeshed with that*.” This sense of self-reflection being used to improve practice was shared by P4, as they described how self-reflection helped them to target their behaviour more effectively in the face of limited time in their current role:

It was a period of self-reflection at looking at, okay, so there are more consults coming in and more requests for work than I can actually provide. So, trying to figure out what’s going to be most meaningful, and what can I contribute most meaningfully.

In a more personal sense, self-reflection was also discussed as a means for preventing burnout whilst maximising the positive experiences of work. Whilst this was similar to the concept of emotional containment of self-reflection in the moment, this was spoken about more broadly in the context of burnout as an experience, rather than the transient emotions and cognitions within a session. P11 spoke to the way in which deliberate, retrospective reflection can foster this:

It’s also that prevention. Because I … think … if there’s an absence of self-reflection and stopping and pausing that … other things can just build up … within you … like … emotional reactions to your work … how you’re handling things. Maybe it’s not in the most efficient way for yourself or your client. And so then that can kind of build up and lead to … burnout … I think it’s important too, I guess it’s that process stuff. It’s not the content but the process of … reflecting, like … are my professional boundaries within my workplace the most helpful for me? Because, you know, if they’re not, then that can lead to more unhelpfulness. So I think that that’s like a really important aspect of self-reflection.

This was echoed by P5 when discussing the value of rating their feelings of burnout using a scale in group supervision:

that’s also useful in terms of prompting, what do I need to do on self-care? How am I feeling in the current climate of whatever’s happening at work? Is there anything I need to do about that? How do I need to address that? So I think it helps me make sure that I’m my best self for my work as well as developing me in areas that I want to as a psychologist and to get better at what I do.

This also overlaps with the contributions from other participants around self-reflection assisting with professional development choices, suggesting a link between fostering continued wellbeing as well as developing as a practitioner. P10 not only elaborated on just burnout but described how self-reflection can mediate some of the challenges of work in order to increase the positive experience: “*And joy too … to actually get some enjoyment in the process, because it’s a very – it’s a great honour, I think … it’s a privilege in some way, but hard work as well*.” P3 described how self-reflection served to address “*perfectionism*”, in that self-reflection had provided them with an understanding of the event and a subsequent plan: “*in that instance, I feel like what I did was appropriate, and now I have an idea of … what we will do the next time they come in*.” P12 also noted how self-reflection was a part of their goal to reduce thoughts and feelings of imposter syndrome: “*a lot of my self-reflection is trying to get better at that anyway, of not doing that*.” There appeared to be a crossover of the personal and professional self, in that self-reflection could be a tool for meeting goals around broader personal cognitive and emotive patterns, such as reducing thoughts and feelings related to perfectionism and imposter syndrome.

There was also a sense shared by clinicians that self-reflection can assist with reflections related to the role of a psychologist, including both how to manage workloads effectively and also broader career reflections. For example, P10 described:

kind of taking stock kind of reflection. What have I been doing? Where do I want to go? Has this been in line with all my values, and … what I want to be doing in life and all that, that kind of reflecting on the role of the psychologist.

This suggests that psychologists are also integrating their own personal values into self-reflection as they consider their role and career more broadly, with this then shaping the outcome of reflection. Overall, there was a sense of personal and professional selves interacting within self-reflection, in that self-reflection is triggered by cues relevant to the motivations and goals of practitioners, whether that is specific to practice or also crosses over into personal wellbeing.

## Discussion

The present study sought to expand on the body of literature focused on self-reflection by exploring psychologists’ experience of self-reflection, including both the process and barriers. Reflexive thematic analysis allowed the researchers to utilise their own knowledge and understanding of self-reflection, facilitating both identification of metacognitive themes and synthesis with existing models of self-reflection as relevant. The overarching theme of interaction of personal and professional self included subthemes of 1) attentive and responsive to cues, 2) personal and contextual barriers to self-reflection, 3) intentionally overcoming barriers, 4) meeting immediate motivations and 5) reflection for learning. The findings are perhaps best summarised by P8, who aptly metaphorised self-reflection as a “rubber band”, in that whilst self-reflection is a vehicle for growth, this stretching can also be uncomfortable for practitioners in a personal and professional sense. This metacognitive understanding of self-reflection – in terms of recognising and sometimes challenging the thoughts, feelings and outcomes experienced throughout self-reflection – was pervasive in the research. This overlaps with conceptualisations of self-reflection that emphasise metacognition (e.g., Knapp et al., 2017), and Merkebu et al’.s (2024) definition of metacognitive reflection, which includes a constant examination and questioning of the process of self-reflection

Overall, participants shared a process of attending and responding to salient cues, monitoring and overcoming barriers to effective self-reflection through the implementation of cognitive strategies, and using self-reflection as a tool both in session and for broader learning. Attending to cues, generating judgements and insights and then enacting behaviour change are common components of models of reflective practice (Lilienfeld & Basterfield, 2020). This process features in Kolb’s (1984) experiential learning theory, Gibbs’s (1988) reflective cycle, Lawrence-Wilkes and Ashmore’s (2014)mnemonic REFLECT model, Grimmer’s (2022) cloverleaf model and Zimmerman and Campillo’s (2003) model of self-regulated learning. Further, the overarching theme of intersection of personal and professional self aligns with the Personal Practice [PP] model (Bennett-Levy & Finlay-Jones, 2018), in particular the “reflective bridge” which integrates self-reflection and learning related to the self and the professional. However, it is also possible that the sample was biased towards psychologists with a richer experience of self-reflection as personal and professional, given participants self-selected to be part of research on self-reflection.

Further, the research mirrored theories of attention and motivation, such as goal-directed behaviour, which notes the role of knowledge and goals in directing attention to stimuli (Corbetta & Shulman, 2002). Overall, there was a sense that the process of self-reflection, which is inherently responsive to both cues and barriers, is both metacognitive in nature and guided by motivation. The hybrid deductive/inductive approach allowed for a balance between participant voice and answering calls to further include the body of literature in the psychological sciences for our understanding of self-reflection (Lilienfeld & Basterfield, 2020) and to begin to understand the commonalities and differences between reflection in the moment and in retrospect Eva and Regehr (2005).

### Implications

The present qualitative study adds insight into not just the observable components of self-reflection, such as behaviour change, but also the metacognitive processes at play for psychologists pertaining not just to their work but to themselves as both practitioners and people. Earlier models of self-reflection describe a rather analytical approach to self-reflection, a problem-solving model of sorts, wherein a problem is identified and then sought to be addressed through some form of behaviour. Whilst participants clearly articulated similar processes both when in situ and after the fact participants also described experiencing – and deliberately employing – complex cognitive, emotional and behavioural strategies that interact with this more analytical problem-solving approach.

The present study suggests that it is possible for psychologists to employ a number of strategies to increase their engagement in effective self-reflection. Importantly, it is also likely that these strategies overlap with their existing procedural and declarative knowledge in that they are likely able to identify thoughts, feelings and behaviours and have an awareness of what strategies may be helpful accordingly. This suggests that self-reflection may be enhanced through greater metacognitive awareness of how one engages in self-reflection most effectively. This approach may also be helpful for supervisors in guiding supervisees in self-reflection.

The present study opens the possibility for further understanding of the links between self-reflection and wellbeing, in particular with burnout. Participants were open in describing the cognitive and affective challenges related to engaging in self-reflection. The *International Statistical Classification of Diseases and Related Health Problems 11th Revision* (World Health Organization, 2024) describes the phenomena of work-related burnout as including “1) feelings of energy depletion or exhaustion; 2) increased mental distance from one’s job, or feelings of negativism or cynicism related to one’s job; and 3) a sense of ineffectiveness and lack of accomplishment” (World Health Organization, 2024, “Burnout” section). Metacognitive awareness of self-reflection may facilitate psychologists being able to self-identify symptoms of burnout, particularly negative affect, cynicism and low self-efficacy. The present study also explored a number of strategies employed by psychologists to overcome these barriers and demonstrated the way in which self-reflection can be protective for mental health through enhancing the positive emotions found in work.

### Limitations and directions for future research

Whilst the present study drew upon the insights of psychologists with a broad range of years of practice, it did not delineate findings based on experience. As a result, a conceptualisation was formed of self-reflection across the developmental trajectory. Further research as to how psychologists at different points in their career trajectory conceptualise and experience self-reflection is warranted, as this may integrate with models of therapist development (e.g., Rønnestad & Skovholt, 2003) and their application to supervision (e.g., Stoltenberg & McNeil, 2010), which suggest that supervisors may tailor supervision in response to developing skill and knowledge acquisition. If we consider self-reflection to be a skill and concept through which to build metacognitive knowledge, as the present study suggests, it follows that supervisors should understand supervisees’ current capacity for self-reflection in order to best tailor supervision. For example, it has been hypothesised that reflective skills are developed initially as task-based reflection, then as self-reflection, and then finally as metacognitive reflection (Granville & Dison, 2005). It has also been hypothesised that capacity to engage in reflective retrospection precedes the capacity to engage in reflection-in-action (Bennett-Levy, 2006). Further research may focus on the role of self-reflection in supervision, including identifying particular barriers, and strategies to overcome such barriers at different levels of reflective skill, as this may assist supervisors in meeting supervisees’ needs.

Whilst the present study sought to explore self-reflection for psychologists, it is possible that findings may be transferable to other health disciplines familiar with self-reflection. Participants spoke of using their own therapeutic knowledge and language with themselves, and it is possible that this may be more unique to psychologists and other health professionals working within therapeutic interventions (e.g., mental health nurses and social workers, psychiatrists) compared with practitioners who work in other settings where mental health intervention is not a typical part of their role (e.g., pathology or cardiology nurses, dentists). Interdisciplinary research on self-reflection may be a valuable area for future research.

Further, whilst a key theme focused on introspection and the value this afforded clinical practice, the research is limited in that this is the perspective willingly shared by participants. In their review of the limitations of the reflective practice literature, Lilienfeld and Basterfield (2020) noted the possibility of the “introspection illusion” (Hansen & Pronin, 2012, p. 346). This involves the value of introspection in addressing biases being overstated and, as a consequence, hindering individuals’ capacity to identify and challenge biases in other ways, leading to an inflated confidence in the objectivity of their decision-making (see Hansen & Pronin, 2012, for a review). Whilst psychologists reported strong merit in self-reflection, it is unclear whether this is indeed evidenced in clinical practice or may, in fact, hinder decision-making.

## Conclusion

When engaging in the “rubber band” of self-reflection, psychologists have shared that they experience and monitor complex cognitive, emotional and behavioural processes, relating to both the personal and professional self. These findings offer practitioner psychologists’ voice to the current research on self-reflection and, in doing so, provide greater depth of understanding regarding the metacognitive processes involved. Further, the findings suggest that there is overlap with psychologists’ experience of self-reflection and models of self-reflection that highlight a process of attending and responding to cues, generating insights and enacting behaviour change. In addition, psychologists shared experiencing barriers to self-reflection and using strategies to overcome these. This is a novel component of self-reflection that is not explicitly identified in existing models of self-reflection. This study has implications not just for practitioner psychologists but likely also for other health professionals who have an interest in self-reflection and reflective practice, and provides support for the continued integration of self-reflection into training and development programmes.

## Data Availability

The data that support the findings of this study are available from the corresponding author upon reasonable request and subject to ethics approvals.
